# Supporting the creative industries through the AI turn: A comparative analysis of Scottish policy and the needs of Scotland’s creative practitioners

**DOI:** 10.1371/journal.pone.0340255

**Published:** 2026-02-19

**Authors:** Suzanne R. Black, Caterina Moruzzi, Nicola Osborne, Melissa Terras, Frauke Zeller

**Affiliations:** Design Informatics, University of Edinburgh, Edinburgh, Scotland, United Kingdom; University of Exeter, UNITED KINGDOM OF GREAT BRITAIN AND NORTHERN IRELAND

## Abstract

In the creative industries (one of the largest UK sectors, which operates at the juncture of business, technology and the arts) creative practitioners are not only consumers of Artificial Intelligence (AI), but also its developers, originators and innovators. This has material consequences for how creative work is performed, valued and understood, as well as global consequences for business structures, the environment, equality and power discrepancies. We take Scotland as a case study for how governmental policy addresses the challenges of AI for the creative industries by comparing Scotland’s Artificial Intelligence Strategy with data gathered from grass roots creative industries practitioners in Scotland through workshops and a survey. We identify differences between the priorities of Scotland’s Artificial Intelligence Strategy and the creative industries, as well as a lack of advice specific to the needs of the creative industries, and offer recommendations on how to put creatives at the heart of AI developments.

## 1. Introduction

Artificial Intelligence (AI) is not new; the worldwide attention that generative AI has been garnering since 2022 is new. As recently as 2020, the Creative Industries Policy and Evidence Centre judged ‘[d]irect applications of AI in creative industries’ to be ‘relatively small scale’ [1], but since late 2022, the arrival of widely available generative AI tools has prompted all sectors to consider incorporating these technologies, and the ramifications of doing so. However, the increased prevalence of AI technologies has specific consequences for many of those working within the creative industries, defined as those industries ‘based on individual creativity, skill and talent, or which have the potential to create wealth and jobs through the development or production of intellectual property’ [2].

AI is a floating signifier that has come to mean different things to different people in different contexts and therefore requires some explanation of its use in this article. AI, in its guises as machine intelligence and expert systems, has been a field of study since its inception in the 1950s. It is used across many sectors, such as in medicine to diagnose diseases [3], in finance to detect risk and fraud [4], and in manufacturing for modelling, analysis and automation [5]. Generative AI is a subset of AI technologies that requires large amounts of training data amassed from existing troves of work (text, images, video, music), creative or otherwise, which are used to generate nearly infinite new variations and derivations of these using tools such as ChatGPT, DALL-E, Sora and SOUNDRAW. The newly widespread awareness and prevalence of generative AI brings with it a set of issues faced by those creative practitioners who willingly engage in using AI technologies, as well as those who are affected by changing perceptions around creative work. Furthermore, the development of AI tools can in some cases be seen as a product of the creative industries since the UK Government’s definition of the creative industries includes the sub-sector IT, software and computer services, and the Scottish Government’s definition includes software and electronic publishing [6]. Beyond the creative industries, AI brings potentialities that all industries must face. Those potentialities extend to a combination of related material and ontological factors: the possibilities introduced by generative AI challenge understandings of art, creativity, originality and even humanity, while the practicalities required to consume and generate content on such a large scale has repercussions for the natural environment, the information environment, and legal, administrative, and economic practices around creative labour. While we have separated these issues into those with ontological or material consequences for those in the creative industries for the purposes of our investigation, it should be clear that all these issues are interrelated.

The Scottish creative industries is a sizeable sector contributing more than £5 billion to the Scottish economy every year [2]. The creative industries is a heterogenous sector, including long-established practices such as traditional forms of craft, textile work and music as well as contemporary technologies for the creation of digital music, art and media, and cutting-edge videogames and other software. Those who participate in the sector also range from individuals and small and medium-sized enterprises to international conglomerates operating in very local spaces or internationally. The Scottish Government estimates that the Scottish creative industries is made up of over 15,000 businesses employing more than 70,000 people as well as many freelancers, and it also includes the number of students studying creative courses [6]. The recent AI turn is affecting those within the creative industries in different ways, depending on the creative role or practice undertaken, and whether newly available AI tools are embraced or rejected. In many cases, creative practitioners are not merely affected by the introduction of generative AI but are active developers, originators and innovators of these technologies.

Those working in the creative industries are both affected by the recent AI turn and in some cases act as active developers, originators and innovators of these technologies. Scotland has a concentration of AI activity spanning from the 1960s to the present-day thriving technology, start-up and AI community across the creative and technology industries. Focusing on Scotland provides a useful lens through which to consider how AI is affecting creatives and their practices in the context of the Scottish creative industries’ specific actors, dimensions, and activities as well as its policy-making and legal system. However, the development and uptake of AI technologies and their consequences are a global concern with numerous effects, including the life and working conditions of people all over the world, the environment, international supply chains, and international legal and regulatory debates, which Scotland’s creative individuals, organisations and companies must navigate.

To explore the potential for using AI and machine learning– a common subset of AI that uses computers to identify patterns and therefore perform a specific task – in the creative industries, we designed the Creative Informatics Creative AI Demonstrator Project. The project, as part of Creative Informatics, was delivered by the University of Edinburgh in partnership with Edinburgh Napier University, Codebase and Creative Edinburgh [7]. We wanted to find out the concerns that various actors in the creative industries had about the introduction of AI and in particular generative AI technologies, and what might be done to alleviate these concerns and enable innovation in this space, particularly in Scotland. To achieve this, throughout 2023, we reached out to creative communities across Scotland to connect with practitioners at different stages in their engagement with AI through a series of workshops and a survey. Here, we compare Scotland’s Artificial Intelligence Strategy, which overarches all areas of economy and society and was published in 2021, with the needs and concerns of Scotland’s creative industries, to determine the driving priorities of each, and to uncover where additional support is needed. Taking this case study approach, this article seeks to answer: 1) the concerns of members of the creative industries about the adoption of generative AI; 2) how the priorities of creative practitioners in Scotland, as ascertained from our engagement with creatives participating in our workshops and survey, map onto the priorities displayed in Scotland’s Artificial Intelligence Strategy; 3) what interventions can be made to best support different segments of the creative industries. We demonstrate that while the concerns of those working in the creative industries broadly align with general concerns about AI, segments of the creative industries have specific needs that must be addressed to protect existing activity and promote innovation. (See our white paper [7] for a fuller discussion of our recommendations.)

## 2. AI and creativity

The ‘creative industries’ is a widely used term that gained currency following the UK Labour Government’s 1998 *Creative Industries Mapping Document* [8 p.3]. The term has since been critiqued for pushing the culture industry towards a neoliberal co-option of creativity into capitalism [9] with an emphasis on individualism and self-entrepreneurship rather than communal values [10]. The creative industries’ conception of creativity is a force leveraged for monetary value – as well as cultural and social value – where IP and copyright are the means by which creativity is currently valued and compensated. For the purposes of this study, we work within this conception to define creative workers and practitioners to be those individuals who are involved in work based on individual creativity, skill and talent in any capacity, whether in paid employment, as freelancers, educators, researchers, or making creative work that is not remunerated. The prevalence of generative AI challenges the fitness of the mechanisms of IP and copyright for fairly determining the monetary value of creative work and who deserves recompense as well as contributing to the debates around creativity, the commons, self-interest and capitalism. As Kate Crawford acknowledges, AI is not simply a technology nor is it autonomous, but rather it ‘is both embodied and material, made from natural resources, fuel, human labor, infrastructures, logistics, histories, and classifications’, which ‘are embedded in social, political, cultural, and economic worlds’ [11]. The consequences of generative AI for individuals in the creative industries are therefore both ontological and material, affecting how creativity is understood and the labour conditions of creative workers.

### 2.1. Ontological factors related to creativity and AI

In computer science, ontology refers to a shared conceptualisation of objects, concepts and other entities [12 p.4] and, from a more philosophical perspective, ontology determines the frames limiting our understanding of the world [13]. The concept of creativity is a shared conceptualisation that underpins how we understand the human capacity to bring knowledge and artworks into existence and generative AI offers a challenge to our understandings of creativity and creative work. Creative work is often valued for its originality and novelty [14] with creativity positioned as being uniquely human [15 p.1]. Recent work into understandings of creativity produced by human and non-human entities – in this case artificial systems – reveals that the prevailing judgement is that humans can perform creativity because they have agency while technological systems based on AI architectures cannot because they are seen to lack agentive capacity [14]. Moreover, challenges to the idea that creativity is a defining human characteristic can lead to resistance, fear and anger [16 p.189].

This division between human and machine-generated content leads to relative judgement over which is the more creative, original or unique, and therefore which is the most valuable. Those who have the most at stake in this distinction are creative practitioners who value creative labour both artistically and in terms of the financial remuneration it can receive, and those who hold the IP and copyright of creative works. The threat to the latter can be seen in the major lawsuits being brought to AI companies in 2025 by Disney and Universal [17], the New York Times [18] and multiple authors [19]. As well as these practical and legislative concerns, there are wider fears about what AI-generated materials will do to the information environment. Renegotiating our understanding of creativity away from this has existential and material consequences for how we value creative work and products. When considered in tandem with the possibility for generative AI to replace the roles of human creative workers, it is of existential concern to the creative industries.

### 2.2. Material factors related to creativity and AI

Many of the global issues pertaining to generative AI have specific material relevance to the creative industries. As well as posing challenges to data use, IP and copyright, and the information environment, AI has devastating effects on physical environments and social justice, generating numerous ethical considerations on a local and global scale. Generative AI requires training data – data on which to base models and decision-making approaches – in order to function. This data is harvested from previously digital and digitised human output, a shared resource known as the digital commons [9], however, those responsible for that output are not consulted about their work being used as training data. Rather, the digital commons may be degraded by an influx of AI generated material and capital flows only to the AI companies rather than creative workers [20]. OpenAI (creator of ChatGPT) has been accused of mass copyright infringement [21]. Copyright is supposed to safeguard creative work but the fast pace of AI growth has left legal reform around copyright and IP reform around digital asset classes struggling to keep up [22].

While the problem of technology companies taking the world’s data to train their AI algorithms has been much discussed [23], the result of this is that a wealth of AI-generated data now exists. There are known practical problems with the entanglement of AI-generated and ‘authentic’ data, where training AI models on AI-generated data leads to the deterioration of those models [24] and with the limits of generative AI to produce new outputs instead of copying existing ones [25]. Excluding deliberate attempts to use AI-generated content for misinformation and disinformation purposes [26,27] this has led to a situation where the internet is home to masses of low quality data, referred to as “AI slop” [28 p.1], which is a threat to our information environment. There are also fears amongst creative practitioners that a surfeit of AI-generated creative content will lead to the production of homogenous output where everything looks the same and the characteristics we use to assign artistic value – creativity, originality, novelty – will be diluted [29,30]. Moreover, creatives who already struggle to market what makes them and their work special might be edged out by more prolific and more visible AI-generated work trained on those very creatives’ data [31].

These can all be understood as ethical problems: the ethics of companies taking data from the commons to train AI models, the ethics of evading IP and copyright legislation, the ethics of polluting the information environment with AI-generated content, and the ethics of creating machine learning models that serve those in power to ‘reproduce, optimize, and amplify existing structural inequalities … to increase profits and centralize control for those who wield them’ [11 p.211]. Not limited to the creative industries, there are the ethics of diminishing the world’s finite natural resources and in the process exploiting workers in the poorest areas of the globe. AI, and the processing of large-scale data in AI systems, has a significant environmental impact, and this is not distributed evenly throughout the world. Despite the existence of AI applications that claim to assist sustainability [32], ‘[d]ata centers are among the world’s largest consumers of electricity’ [11 p.43].

These concerns have an effect on the way certain creative jobs are valued and whether those responsible for creative work used to train AI are fairly compensated for their labour. A report from Microsoft that analysed usage of its generative AI chatbot, Copilot, found that users’ activities overlapped with several creative roles. The top forty roles that they designated as being most at risk from a generative text tool included proofreaders, writers, authors, journalists and editors [33]. Meanwhile, AI-generated images and visuals have been seen as a threat to graphic design roles [34]. There is a worry that any creative role that already involves the creation of digital media is at risk of being partially replaced by AI agents, despite criticisms of the quality of output from current AI technologies [35,36]. These roles can be found in many of the industries that make up the creative sector, such as advertising, design, photography, film and video, computer games, radio and tv, writing and publishing, and software or electronic publishing. Microsoft’s list of the forty roles that they designated as being least at risk from a generative text tool does not include any specific creative roles but it does include multiple trades and roles that are crucial to the creative industries, such as floor sanders, pavers, roofers, cement masons, painter/plasterer assistants, and production worker assistants, all of which are roles that can contribute to creative industries including building visual art or fashion exhibitions and installations, and film, tv, music and live performance sets, stages and studios [37]. These predictions about the roles that AI may partially or fully replace are those that are typically associated with knowledge work and human creativity while the roles assumed to be safer are often categorised as less creative, as vocational, practical and physical roles that are nonetheless critical to the creation and delivery of certain creative products, contributing to the argument that the tasks being outsourced to computer technologies are the very ones that are seen as essential to humanity [38].

A situation in which all creative work is taken from the commons by a small number of tech companies, combined with proprietary algorithms and then turned into a commodity, devalues multiple types of creative work and creative jobs materially. Questions are ongoing over who gets to claim ownership of and any related revenue streams associated with creative work generated using AI tools or generated as the result of large uncredited data sets [39] and this is complicated by the variance in copyright laws internationally. The introduction of AI to replace creative work is part of a longer trend of the devaluing of creative roles [40,41].

The potential harms related to generative AI and issues of data and IP affect both the creative industries and wider society. These extend to a loss or devaluing of creative jobs, the theft of intellectual property by large AI companies and, more widely, the incorporation of bias and representational harms into decision-making systems. Creative jobs are already being replaced by AI tools in the areas of language translation [42] and copywriting [39] while the human-generated data that these replacement tools are built upon are taken without compensation. There is a feeling that ‘[w]e need to protect our intellectual property, and we need to limit the ability of generative AI developers to freely plunder our minds without permission or compensation’ [21].

Issues of IP are related to ethical considerations and fair compensation for labour. There is the ongoing challenge of differing definitions and case law (not only on AI but also related areas of data protection and privacy) across different national and regional legal systems. Calls for better technologies around digital provenance, that is ‘the use of metadata to present information to an end user about the origins of, and alterations to, a piece of digital content’ [43 p.2], have been made but are difficult to implement in practice.

Bias and representational harm arise from the ways in which training datasets for generative AI tools are sourced and labelled, and how AI algorithms function to generate plausible content. The opaqueness of AI companies’ algorithms makes it difficult to investigate such claims, but efforts to reverse engineer how the algorithms of some of the most popular generative AI tools work have found that they reproduce normative identities and narratives [44] so that text generation tools (OpenAI’s ChatGPT, Google’s Bard (now Gemini) and Microsoft’s Bing AI) were found to ‘tend towards being generic, centrist, normative, and banal’ [44 p.10]. When the text-to-image tool Midjourney is asked to generate images of queer people it returns stereotypical images of those in LGBTQIA+ communities [45] and struggles to generate images of Black doctors treating white children, although it has no problem generating the opposite [46].

These ontological factors – about the value of creative work and the creative industries – and material factors relating to the human workforce, sustainability of planetary resources and seismic changes to the information environment are interlinked. As Kate Crawford acknowledges, AI is not simply a technology nor is it autonomous, but rather it ‘is both embodied and material, made from natural resources, fuel, human labor, infrastructures, logistics, histories, and classifications’, which ‘are embedded in social, political, cultural, and economic worlds’ [11 p.8 & 211]. Generative AI prompts us to rethink ideas such as creativity, originality and authenticity, and ask questions about the value of creative labour, which then manifest in the material conditions for those working in the creative industries.

### 2.3. Creativity and technology

Technology and art have always existed together, with technology taking the role of enabling artistic innovation while conversely being cast as a threat. The introduction of photography in the nineteenth century sparked debates about the role of technology in art, as did the introduction of digital software for editing images, such as Adobe Photoshop, in the 1990s and yet, painting and photography still persist decades after their demise was predicted.

New technologies challenge our understanding of creativity if we stick to the definition given by the Scottish Government Culture and Major Events Directorate of ‘*individual* creativity, skill and talent’ [2]. There are multiple ways to define authorship with different legal and governmental framings privileging different ones. In the US there is a legal rule that copyright can only be awarded in the case of human authorship [47] while in the UK there is some provision that allows for computer generated work, without human contribution, to be awarded copyright (and that copyright goes to the facilitating individual or organisation), which is being disputed [48]. Running converse to the idea that creativity is ‘uniquely, even sacredly human’ [49] is the idea that creativity is ‘combinational’ [50] and something that can be ‘synthesized, amplified, and manipulated’ [51], which recombines existing pieces in new ways [16 p.193].

This understanding of creativity as relying on a tradition of pre-existing ideas and artworks along with the willingness to open up authorship to include human and non-human actors, such as technologies and tools, leads to a redefinition of creativity as a relational activity that arises from networks of people and technologies. Celis Bueno et al. go further to renegotiate creativity in the context of generative AI, arguing that getting away from seeing it as something uniquely human instead reconfigures it as arising from ‘the interactions between technologies, practices, and social arrangements’ [15 p.6]. This reframing draws upon Leah A. Lievrouw’s description of the confluence of social and material factors in communications media [52] where ‘Lievrouw’s diagram of mediation allows for a materialist analysis of the network of relations between generative AI technologies, the creative practices that make use of these technologies, and the socio-economic imperatives behind their design and implementation’ [15 p.6]. Under this redefinition of creativity as something that has multiple components, of which humans are only one, generative AI challenges the model of the primacy of human creativity and instead implies a ‘conceptualisation of creativity that privileges neither human users nor machine learning algorithms but instead emphasises a relational and distributed form of agency’ [15 p.11]. This model is echoed in Caterina Moruzzi’s model of distributed creativity and authorship where humans and machines work together as part of a network and neither operates alone [14].

Evidence that we are already theorising creativity in the context of generative AI as being networked can be seen in claims that ‘artificial intelligence is both embodied and material’ [11 p.8], that ‘[a]lgorithms are not simply or originally machinic’ [53 p.10], and that ‘computer algorithms’ can be construed as ‘a moment of dialogue between human and machine’ [16]. Celis Beuno et al. remind us that human and automated labour are not necessarily distinct, since generative AI requires (often hidden) human labour for ‘new tasks such as data annotation, data moderation, and model testing’ to give the illusion of ‘AI automation’ [15 p.9]. Rather, global mechanisms of extractive labour are required to perpetuate the existence of generative AI tools.

### 2.4. Policy interventions related to creativity and AI

Some concerns arising from AI, such as data use, IP, ethics, labour conditions, and an impoverished information environment, may be mitigated by regulation and policy. Scotland has been pro-active in creating and implementing an AI Strategy. Scotland being part of the United Kingdom is a devolved nation and as such responsible for Scotland specific matters, including the economy, education, health, justice, environment, equal opportunities, and consumer advocacy and advice [54], all of which have been or will potentially be affected by AI. The Scottish Government also has its own Artificial Intelligence Strategy, which differs from the UK and European strategies. Published by the Scottish Government in March 2021, Scotland’s Artificial Intelligence Strategy [55] is delivered by The Data Lab, which helped write the Strategy, and by the Scottish AI Alliance, which is creating and maintaining The Scottish AI Playbook. At the highest level, it calls for ‘trustworthy, ethical and inclusive AI’ [55 p. 5] and links these to Scotland’s National Performance Framework, which aims ‘to grow wellbeing and economy in Scotland while reducing inequality’ (p.8) and the principles of the Organisation for Economic Cooperation and Development, which are: to benefit people and planet, respect law, transparency, continued attention to security, and accountability (p.20). The Strategy document describes practical steps to achieve this. These include ‘developing a skilled and diverse workforce, supporting organisations to be innovative and providing the right investment… by creating the necessary skills, data infrastructure and access to funding, as well as influencing national and international policy and regulation’ (p.34). In addition, the Strategy includes prompting the public to become more educated about AI (p.34) and aims to help mitigate the environmental impact of AI by encouraging the creation of green data centres (p.26).

The UK Government and European Union also published AI strategies with differing priorities. The UK and EU contexts are relevant for any appraisal of Scotland’s Artificial Intelligence Strategy as some areas of Scottish law and policy-making are reserved and therefore set by the UK government, and Scotland remains mindful of EU nations as trading partners so their AI Strategy has specific relevance for export and trade in Scotland.

The UK National AI Strategy, published in September 2021 and updated December 2022, focuses on how the UK can lead the world in a ‘progressive regulatory and business environment’ [56]. One of the ways the UK government plans to enact this is by the establishment of the AI Standards Hub, led by The Alan Turing Institute in partnership with the British Standards Institution and the National Physical Laboratory. The Hub aims to set global standards for AI that ‘consider lighter touch options, such as guidance or voluntary measures, in the first instance’ to ‘build the most trusted and pro-innovation system for AI governance in the world’ [57]. The two priorities that emerge from the UK’s Strategy are consumer confidence and creating the conditions for commercial innovation.

The European AI Strategy seeks a balance between safety and innovation ‘aiming to boost research and industrial capacity while ensuring safety and fundamental rights’, delivered through a legal framework on AI and a Coordinated Plan on Artificial Intelligence [58]. In this approach, there is a focus on the importance of rules to identify, address and monitor ‘high risk applications’ of AI [59]. The Scottish, UK and EU strategies demonstrate different priorities: while the Scottish government focuses on social justice, the UK has focused on enabling economic growth (re-emphasised in January 2025 announcements [60]), and the EU on balancing the rights of citizens with rules and regulations.

## 3. Methods

To compare the needs of creatives against the Scottish Government’s high-level Strategy we canvassed opinions from a range of creative practitioners working in the context of expanding AI technologies. We used a case study methodology, which enables the investigation of ‘a phenomenon in its real-life context’ via multiple methods of data collection [61 p.95]. This involved a series of workshops as a means of ‘creative problem solving or innovation in relation to a domain-specific issue’ [62 p.71] with participants from across Scotland who use AI in their creative practice, those who are interested in doing so, or those who otherwise work in or adjacent to the creative industries. The workshops attracted 64 participants who were curious about AI and what it might mean for their creative practice, but were generally not very experienced in using AI technologies. We also created a survey, running concurrently, designed to facilitate responses from a wider range of participants. The survey did not attract as many responses as we expected (only 30 after filtering for bots and mischievous respondents), but these turned out to be detailed and reflective responses from creative practitioners experienced with using AI technologies, which worked well to complement the data gathered in the workshops.

Research ethics approval was applied for and granted by Edinburgh College of Art (ECA), University of Edinburgh, for work as part of the AHRC Creative Informatics project. [For the approved form, please see 63]. For survey data, written consent was obtained from all participants before they provided any data. The recruitment period for the survey was 19 June until 30 September 2023. For workshop data, participants were made aware that their contributions would be used in research outputs but individualised consent was not sought as data were analysed anonymously. The recruitment period for the workshops was 1 July to 16 November 2023.

### 3.1. Workshop Design and Implementation

We held three in-person workshops (on 10 August, 7 September and 16 November 2023) with a total of 64 participants. Participants were recruited through Creative Informatics’ established communication channels – internal mailing lists, newsletters and social media accounts (Twitter – now X, LinkedIn) – as well as contacting creative and technological organisations in Scotland and asking them to publicise the workshop, including the Scottish AI Alliance.

In the workshops, participants were asked, in groups, to design an AI tool and think through the resources required to realise the idea, in order to prompt participants to think through creative use cases for AI, the necessary dependencies, and the potential consequences. Participants were asked to record their responses on sticky notes. The sticky notes were photographed in situ and later typed up. The resultant data were analysed via thematic analysis using an inductive, latent, critical and relativist constructionist approach [64 p.10].

### 3.2. Survey design and implementation

The survey contained questions designed to gather countable data and free text responses informed by literature on how AI is used, understood and designed to find out how the participants were already using AI technologies as well as the support and resources needed to take this work further.

We delivered the survey on Jisc Online Surveys, a Data Protection and GDPR compliant survey research tool. The survey was distributed through Creative Informatics’ established communication channels to reach potential participants within CI’s community and further afield: internal mailing lists, newsletters and social media accounts (Twitter – now X, LinkedIn). The survey contained 46 questions. A total of 85 participants completed the survey. Of the 85 participants, 55 were excluded due to giving bot-generated or otherwise bad faith answers [7] leaving 30 responses to be analysed, which is within the expected range for a typical qualitative online survey [65 p.649]. For a list of the survey questions, please see S1 Appendix.

We analysed the qualitative questions in accordance with the principles of reflexive thematic analysis [64], which eschews quantifying the responses and providing frequencies in favour of a more subjective approach.

### 3.3. Participants overview

We asked the participants about their age, ethnicity, gender and sexuality to gain an overview of how diverse our cohort was in relation to the population of Scotland. Small numbers of participants have been aggregated to aid anonymity and numbers have been given as percentages to minimise the risk of exposing individuals. The 64 workshop participants were 42% female, 39% male, 16% preferred not to say and 3% had another gender identity. 61% were white, 17% preferred not to say, 11% were Asian or British Asian, 6% belonged to mixed or multiple ethnic groups and 5% belonged to another ethnic group. 53% of participants chose not to disclose their sexuality, 38% were heterosexual, 5% lesbian and 3% bisexual. The participants were predominantly aged between 25 and 54. The 30 survey participants were 50% female, 3% male and 7% had another gender identity. 80% were white, 10% belonged to mixed or multiple ethnic groups, 7% were Asian or British Asian and 3% belonged to another ethnic group. 70% of participants were heterosexual, 17% preferred not to say and 13% had another sexual orientation. The majority of the participants were aged between 25 and 64.

This compares with a Scottish population that skews female, white, aged 65+ and heterosexual from Scottish population data taken from the 2022 census. The participants sampled in this study are therefore broadly in line with the Scottish population in terms of gender and sexuality, if we assume those who chose not to answer those questions follow a normative population distribution, that their ages skew younger than the general population, and they are more ethnically diverse than the general population (with a maximum of 78.1% white participants in the workshops, 80% in the survey, compared with 92.9% for the whole of Scotland). With such a small sample (64 in the workshops and 30 in the survey), achieving representativeness of the creative industries in Scotland is not achievable. The views represented in this study are reflective of a small selection of those working in and adjacent to the creative industries with an interest in understanding the effects of AI on those industries, whether as users of these new technologies or people affected by them.

We collected limited data about the participants’ roles within the creative industries. These extended to practitioners working across graphic design, music, poetry, publishing, photography, ceramics, theatre and the visual arts, as well as students and educators in these areas. Some participants identified themselves as holding adjacent roles as technicians or working in programming or engineering. Some identified themselves as freelancers. Several participants worked for arts or heritage organisations in a managerial or strategist capacity. Many of the participants held more than one of these roles.

### 3.4. Participant experience with AI: Workshops

The workshop participants were asked about their backgrounds. There was a mixture of students, researchers, academics and those involved in teaching and education, and creative practitioners across the areas of graphic design, music, poetry, publishing, theatre and the visual arts, although many of the participants held more than one of these roles.

The workshop participants were asked about their experience with AI to provide context for their concerns and priorities. The majority of the workshop participants stated an interest in AI and in using it in creative/ artistic work (39 out of 64 participants), however, only 12 of the participants stated they already used AI in creative/ artistic work. A further 14 used AI for other purposes. These results signal that the workshop participants were interested in AI technologies but not necessarily experienced in using them yet.

To gain an overview of the participants’ prior relationships to AI, we asked them ‘what does creative AI mean to you?’ and invited them to write as many responses as they wanted on sticky notes. During the workshops a focus on generative AI emerged as, when asked to clarify what kinds of AI technologies they meant, the participants often referred back to text or image generation tools like ChatGPT and Midjourney.

### 3.5. Participant experience with AI: Surveys

The 30 survey participants typically indicated greater experience with using AI technologies than our workshop participants and this generated a different perspective on what AI might mean for the creative industries. 27 of the 30 survey participants indicated that they had little (9 participants), some (13) or lots (5) of experience with AI, implying that the survey participants were more engaged with using AI technologies than the workshop participants.

The types of tools the survey respondents had experience with skewed towards generative AI tools, although many of them had experience with other kinds of AI technologies. The most popular tools were ChatGPT (22 respondents), DALL-E (12), Midjourney (9), Bard (8), Stable Diffusion (8) and GPT-4 (8). This use extended to complex use cases such as developing new technologies, cataloguing data, and creating artworks. Two respondents had not used any AI tools.

## 4. Findings

### 4.1. Workshops

During the design activities in the workshops, participants were directed to think through the real-world practicalities involved in creating AI tools for the creative industries. Their responses coalesced into two main themes: 1) practical concerns around the impact of data and IP on creative work and subsequent consequences for creative jobs, and 2) existential factors related to creativity.

#### 4.1.1. Practical concerns for creative work.

The participants surfaced practical concerns around data acquisition, use and protections. Most of the groups brought up the importance of having access to the required data to train AI models and once acquired, the storage and management of those data, as well as issues of privacy and data standards. Workshop data was gathered according to group rather than individual. The identifier ED3, for example, indicates the quote is attributed to Edinburgh workshop group 3. Survey participants are indicated by the letter ‘S’ and an id number, for example, S15. They raised questions around ‘who owns data?’ (ED3), how to ‘adjust for dataset biases’ (GL3) and activities like ‘labelling and classifying’ (GL4). This was accompanied by the difficulties of applying existing intellectual property and copyright rules to data used in training models and the outputs of those models with participants asking ‘what are copyright implications for AI-gen[erated] materials?’ (ED1) and suggesting that one solution could lie in building AI tools for ‘[i]dentifying forgeries/ derivations’ (ED4). In addition to these concerns, participants raised questions around labour, particularly around ‘compensation for data contributors’ (DU5). Surprisingly, there was only one comment around the risk that AI poses to creative jobs with one group writing that their proposed tool is ‘definitely desirable as a service…but as a production team it would take our role’ (ED5).

#### 4.1.2. Existential concerns for creative work.

The more ontological concerns raised by participants extended to how AI technologies may impact creative processes and understandings of creative value, encompassing questions about the changing relationship between humans, technology and creative output. There was a balance between potential benefits of incorporating AI into creative practices, such as the serendipity of ‘happy accidents’ (DU5) and offloading cumbersome tasks allowing one to be ‘100% creative’ (ED5). Conversely, there were concerns around the ‘validation’ (ED2) and ‘originality’ (ED5) of work created with generative AI input and a fear that this might lead to an ‘erosion of personal agency for users’ (ED4) of such tools. One group floated the critical question ‘can AI be creative?’ (ED1).

### 4.2. Survey

From the survey data we identified three themes: 1) The need for the creative industries to engage with AI; 2) Concerns about AI in the creative industries; and 3) Barriers to using AI in the creative industries.

#### 4.2.1. The need for the creative industries to engage with AI.

This theme reflects a feeling by survey participants that AI is already part of the creative industries and must be grappled with. The participants identified several changes that AI can offer, some beneficial, some less so. Some participants were excited about incorporating new skills into their practice – ‘I taught technology for many years and am very keen to keep up to date with skills and knowledge so that I can apply it to my sculpture’ (S12) – and others felt a ‘need to become familiar and skilled in using it’ (S29). There is a possible split here in motives, with some creative practitioners keen to embrace new technologies and some feeling they have to ‘keep up’ to remain relevant or to stay employable. Several of the participants conceptualised AI as a ‘helper’, especially with idea generation where ‘AI can come up with options that I would never think of, and can also generate 1000s of options almost instantly’ (S1). By far the biggest impact they expect is efficiency. AI is described as allowing the user to ‘reduce mechanic/ boring aspects of creative work’ (S5) and ‘[i]mprove work efficiency’ (S15). Again, there is perhaps a distinction to be made between creative practitioners believing that AI can help their work and feeling that, if the widespread adoption of AI is inevitable, it would be beneficial to find ways to make AI work for them and to minimise disruption to their established practices. This overlaps with concerns around changes to creative jobs, where AI technologies may fit into the creative process, and the pressure felt by those in the creative industries to contend with AI. Some participants saw AI as an opportunity to think through our relationships with technology as it relates to creativity as ‘[o]ften what we presume is inherently human or computational can be applied to both areas’ (S4). This sentiment points to the artificial divide between human and technology-based creative work, which is further discussed in the Discussion section.

#### 4.2.2. Concerns about AI in the creative industries.

Participants identified several key components for working with AI and raised concerns about them. These cover some of the concerns already raised by the workshop participants around data and IP, ethics and consequences for creative workers. One participant summed up concerns around data: ‘My main concerns are: datasets used in machine learning (whether they are representative, what data they contain, how they are annotated); who builds the machine learning systems, who makes the decisions, who has control, and whether diverse voices are represented in these processes’ (S16). This participant covers many of the main concerns around data use with AI technologies, including how datasets and the algorithms that use them can contribute to inequalities and bias that have real-life consequences. The survey participants noted the entangled practical and ethical issues with getting and using datasets for AI tools, finding ‘[e]thical concerns around lack of consent gathered for collecting and using the data that powers AI/ ML technologies’ (S13) and ‘[w]orries about big tech companies accessing copyrighted data when we as researchers spend a long time to obtain permissions for it’ (S3). The survey participants raised additional concerns around labour, expressing the fear of AI reducing the value of creative jobs. One participant argued that ‘people should value human creation’ (S1), appealing to the distinction between human and machine-created content that persists in discourse around generative AI. There was also the worry that ‘it might make certain job roles redundant, and I think that’s inevitable’ (S26). This final quote offers the perspective, however reluctantly, that changes in technologies occur and bring with them attendant changes in society, which may be beneficial to some and detrimental to others.

#### 4.2.3. Barriers to using AI in the creative industries.

When asked about possible factors preventing them from using AI, common barriers identified by participants were: access to tools/ software/ compute, time, funding, training, concerns around privacy, ethics and legality, and having the requisite skills. Suggested tactics to address these were: access to funding for projects, training and large-scale compute; and intervention by government agencies to promote creative industries’ interests in this space. These suggested interventions were used as the basis for the recommendations we provide in Section 6 about how policy, funders and academic institutions can support the creative industries in Scotland to make use of AI tools in the most beneficial way.

## 5. Discussion

In this section, we reflect on the concerns and priorities of the members of the creative industries in Scotland that we consulted regarding the adoption of generative AI. We discuss where their concerns overlap with the wider critiques of generative AI that we introduced in the literature review section of this article, ‘AI and Creativity’. We then examine the ways in which Scotland’s Artificial Intelligence Strategy supports elements of the creative industries in Scotland, before, in the Recommendations section, offering a list of interventions that could be made to better support the elements of the creative industries consulted in this study.

### 5.1. Ontological factors related to creativity and AI

The ways the participants in the workshops and survey conceptualised and debated generative AI relate to deeper arguments about the roles of creativity and technology, how they are defined and valued, and the consequences these have for creative jobs. When the workshop participants questioned how AI technologies could fit into creative processes – whether as a helper or a time-saver – and expressed their concerns over a potential diminishment of human agency in creativity, these fears tap into what it would mean for creativity and creative roles to be redefined and devalued. Although there was only one explicit comment about potential job losses arising from wider AI use, the participants’ conceptualisation of creativity as something algorithmic, non-human and easily replicable could challenge the place and value of multiple roles within the creative industries. The survey participants were more specific about these worries, with participants expressing fears that the devaluing of human creation would lead to the elimination of certain creative jobs. The concerns that surfaced in our study tap into long-running debates around the definition, role and value of creativity in relation to technology, as noted in our literature review.

The challenge that generative AI poses to our understandings of creativity is of huge concern to those in the creative industries in Scotland, and this is not surprising. Technology and art have always existed together, with technology taking the role of enabling artistic innovation while conversely being cast as a threat. The introduction of photography in the nineteenth century sparked debates about the role of technology in art, as did the introduction of digital software for editing images, such as Adobe Photoshop, in the 1990s and yet, painting and photography still persist decades after their demise was predicted. As has been repeatedly emphasised throughout this article, and shown through our engagements with the creative industries via workshops and a survey, it is not possible to separate ontological concerns around generative AI for the creative industries from material concerns. While generative AI implies distributed agency across human and non-human actors, it is only humans in this network that can be hurt.

### 5.2. Material factors related to creativity and AI

The potential material consequences of the adoption of generative AI for creative roles that surfaced in our engagement with members of the creative industries extended to ways data is amassed and used, the suitability of copyright legislation, and the ethical implications of LLMs and machine learning to worsen structural inequalities.

The workshop participants raised open questions around ownership of training data and of the outputs of generative AI tools while the survey participants were more focused on issues of representation, bias and power, recognising that decisions made based on the outputs of LLMs created by a small number of companies can have real-life consequences for a large number of people. This has already been seen when AI technologies are used in criminal sentencing to correct for the biases and inconsistencies of individual judges but only serve to amplify existing biases [66]. While the concerns about job loss are directly relevant to those in the creative industries in Scotland, these wider representational issues created by global AI tools affect all those who operate in an information environment that is being shaped by these technologies. As this article has argued, it is difficult to disentangle the concerns around generative AI: they span data and legal issues around IP, which relate to labour issues around compensation for creative work and fears of job losses, generative AI content affects the information environment in which future creative work is done, and all of these have an ethical dimension. Generative AI brings with it a suite of ontological and material concerns for the creative industries. In addition to concerns specific to the creative industries, there are global implications for the widespread adoption of AI technologies in terms of environmental sustainability.

### 5.3. Suitability of Scotland’s AI strategy

As determined from this case study, the members of the creative industries in Scotland that we consulted identify a series of concerns around the increasing prominence and implications of AI technologies. These coalesce around the material factors of fair compensation for creative work, the regulation of AI companies to safeguard existing creative work and jobs, and the potential bias and representational harms resulting from AI-based decision-making systems. There is also the ontological factor of AI-generated content devaluing creativity in a broader sense. Scotland’s Artificial Intelligence Strategy [55] addresses some of these material concerns. Data use is addressed with plans to draft a list of ‘trusted algorithms’ (p.36) and ethics are invoked by a call for avoiding discrimination (p.20). There are calls for collaborative forms of governance (p.33). Labour conditions are addressed by calls for educational resources for the general public and in the service of ‘developing a skilled and diverse workforce’ (p.34).

While the priorities of both Scotland’s creative practitioners’ and Scotland’s Artificial Intelligence Strategy extend to the regulation of AI, the ethics of AI, mitigating bias and representational harm, and labour conditions, the creative practitioners we engaged with also prioritised intellectual property protections, protecting creative jobs, and maintaining the value of creative work. The Strategy additionally prioritises environmental sustainability.

However, the bigger picture questions around creativity and creative work are not reflected in the Strategy, despite their tangible effects on material conditions. While concerns around a glut of AI-generated material and the potential flattening of artistic styles are a huge cultural challenge that individual governments cannot control, IP and copyright are the mechanism by which creative work is valued and remunerated, and this is within the scope of UK government legislation. The Strategy does not mention IP directly, despite intellectual property rights being the mechanisms by which creative work is associated with economic value. This may be an artefact of making a Strategy in the context of a devolved administration, since intellectual property is one of many reserved powers [67]. There remains a responsibility for the Scottish Government to influence conditions for creatives, especially as funding for sports and the arts is devolved. The aim stated in Scotland’s Artificial Intelligence Strategy to ‘identify the regulatory and financial levers Scotland has to realise the vision [of the Strategy], such as funding and procurement and where we need to influence nationally and internationally’ [55 p.31] likely refers to the requirement for Scotland to work within a wider context of regulation.

From the discrepancies between the priorities of the Strategy and creative practitioners, we can conclude that Scotland’s Artificial Intelligence Strategy does not meet the current needs and priorities of the creative industries, or at least SMEs and entrepreneurs within the creative industries as represented by our research participants. This is likely due to a number of factors, such as the scope of the Strategy and the publication of the Strategy prior to mass engagement and adoption of ChatGPT.

### 5.4. Global Context

The development of AI technologies is of global concern, involving international tech companies and workers on the ground – often in the Global South – who suffer the environmental damage and poor labour conditions required to build microchips and batteries. Governments developing new legislation or updating existing legislation for the context of AI must find a way to balance their own national priorities with ethical obligations to the wider supply chain.

## 6. Recommendations

Taking into account the needs of the creative industries in Scotland captured in our research, we set out a list of recommendations, which we believe will also have wider benefits.

- **Involvement**. There is an appetite in the creative industries for more engagement with AI. Creatives should be supported by policy to take a leading role in AI’s creation, development and implementation (not just as adopters and consumers of AI). This could take the form of consultations by the Government with groups representing all elements of the creative industries, from different sectors, backgrounds and career stages.
- **Education** at all levels: for creatives implementing AI in their work; for institutions and organisations that steward the outputs of the creative industries (such as Galleries, Libraries, Archives, and Museums, as well as publishers, commercial art galleries, etc.) around data provenance and the discernment of generative and/ or deep fake content; for the public as consumers of creative work to allow them to make informed choices. The Scottish AI Alliance already offers a wealth of useful resources about AI, including the free online course, Living with AI, which is designed to better inform the Scottish populace about AI. We suggest further funding of the Scottish AI Alliance to enable them to create tailored educational material for all sectors, including the creative industries, with targeted advice.
- **Support**: in the form of sustained funding for innovative R&D work and scale-up potential with AI in creative contexts, enabling cross-sectoral innovation across SMEs, academia and the expertise of larger organisations (e.g., the GLAM sector) to develop capacity, competency and competitive and sustainable creative industries; access to and financial support for large-scale compute (e.g., the UK’s National High Performance Computing system); the establishment of regional Creative AI hubs for the convening of creative SMEs and freelancers with necessary infrastructure, funding opportunities, training and skills development, and peer support. To achieve this, we advocate for academic/ industry partnership models proven in the Creative Industries Clusters Programme, where significant targeted investment is made into upskilling and supporting individuals, SMEs and R&D-focused organisations by providing financial, business, ethics, and legal support and networking over a sustained timeframe.
- Strong and transparent **governance** on issues within the Scottish Government’s purview, as well as a voice in UK Government and international debates about AI regulation, such as developing intellectual property law. As intellectual property rights are a reserved power and therefore fall under the jurisdiction of the UK rather than the Scottish Government, there is less ability for the Scottish Government to intervene here. The UK Government’s 2024 consultation on AI use, rights and training data in the UK prompted a very strong demand from those who hold the rights to data to be allowed to opt in to it being used by AI companies, rather than having to opt out after the fact [68]. We would like to see the Scottish Government acting wherever possible to safeguard the rights of creatives in line with its ethos of trustworthy, ethical and inclusive AI.
- A commitment to **responsible AI** (see, for example, BRAID UK) to ensure environmental sustainability and social inclusion. The ethical dimensions of AI are the responsibility of individuals, companies and organisations as well as regulatory bodies. An AI charter voluntarily signed by individuals, companies and organisations is one possible way of establishing ethical standards and accountability. We suggest a charter co-created by stakeholders in the Scottish creative industries along with a certification model backed by the Scottish Government to indicate that its core tenets of trustworthy, ethical and inclusive AI are being followed.

While this article focuses on the Scottish context for creative AI, these are technologies with a global reach and many of the key challenges and needs of creatives in Scotland are mirrored elsewhere. This article therefore contributes to a global conversation around the use, regulation and material consequences (for creative workers, for labour forces, for the environment) of these technologies.

## 7. Limitations and future work

One of the limitations of this study is the speed of change in the AI landscape as technologies, legislation and public opinion are quickly shifting. Another is the variety of different understandings of and engagement with AI technologies. We sought to mitigate this by using an expansive definition of AI in our recruitment materials and, upon realising that the majority of our workshop and survey participants were discussing generative AI, focused our study on generative AI in the Scottish creative industries. A third limitation is the small sample size recruited for this study. While we believe that the benefits of sustained engagement and the capturing of high-quality data from a smaller number of participants outweighs the benefits of larger sample sizes, future work could extend reach by employing follow-up interviews to access an even deeper stratum of data and by seeking to reach a larger number of participants, perhaps by partnering with teaching and training organisations.

By engaging with a larger population and being more attentive to the different demographics within the creative industries, future work could profitably extend and nuance our findings to reflect the different attitudes and potential consequences for AI adoption on different professions and job types within the creative industries to yield more granular insights and further tailor the recommendations we have offered. Similarly, another avenue of exploration is the attitudes and potential consequences for AI adoption on creatives at different stages in their careers to uncover links to equity, access, skill and other conditions.

Future work could also look further afield for other governmental responses to safeguarding the creative industries in their national contexts. We were only able to touch on the global context in this article and we are aware that, for instance, legal and governmental interventions in North America (where several key test cases are being heard and the majority of global scale generative AI services are now based and/or financed) can have a significant impact on the global technology sector.

## 8. Conclusions

We sought to compare whether governmental policy adequately addresses the challenges of AI for the creative industries using Scotland as a case study. We compared the priorities of Scotland’s Artificial Intelligence Strategy with data gathered from grass roots creative industries practitioners in Scotland by analysing responses from a series of workshops and a survey. We found that, although some of the priorities of the Scottish Government and the Scottish creative industries align, there are areas in which the needs of the creative industries are not being met. The creative industries in Scotland require specific support, funding, education and governance as well as, crucially, participation in making decisions about their involvement in the direction of travel of AI. They also require protections under law for how creative work is valued and compensated and, although this is outside of the scope of the devolved Scottish Government’s powers, the government needs to be active in advocating for the rights of creative workers at UK and international levels. Based on this analysis, our recommendations provide a framework to improve the capacity of the creative industries in Scotland to successfully navigate the AI turn to put creatives at the heart of AI developments.

## Supporting information

S1 Appendix List of survey questions. (PDF)
