# Supplementary material for: Supporting the creative industries through the AI turn: A comparative analysis of Scottish policy and the needs of Scotland’s creative practitioners
Source: PLoS One. 2026 Feb 19;21(2):e0340255. doi: 10.1371/journal.pone.0340255 (PMC12919769; doi:10.1371/journal.pone.0340255)
Supplement: S1 Appendix — (PDF) [file pone.0340255.s001.pdf]

Creative AI Demonstrator Project

Creative AI Demonstrator Project from Creative Informatics

The Creative AI Demonstrator Project is an AHRC and DCMS-funded project to find out about current work happening and the support needed for future work in the field of creativity and artificial intelligence / machine learning, or ‘Creative AI’.

We define ‘Creative AI’ broadly to include any use of machine learning (ML) systems (including, but not limited to Generative Adversarial Networks (GANs), Large Language Models (LLMs), and speech, image, text and audio recognition and generation) at any stage in the creative process.

We are additionally interested in understanding more about how the music sector in Scotland is using AI / Machine Learning to create new products, services, or content, and are investigating how best to invest resources to support innovation in this area.

We welcome survey participants from both Scotland and further afield.

The survey closes on **Monday 31 July 2023**. To thank you for taking the time to complete the survey, a prize draw will be held following the closing date with the opportunity to win one of five £50 high street vouchers.

If you have any questions about the survey, or would like to withdraw your responses, please email: suzanne.black@ed.ac.uk

For more information about this survey and what it means to take part, please visit https://creativeinformatics.org/creative-ai-survey-information.

Data Protection

In providing your completed survey you are giving explicit consent for us to use this data in our research processes. The data is managed confidentially. Your data will be held by the Creative Informatics delivery team based at the University of Edinburgh, with some data shared with our funders the Arts and Humanities Research Council and the Department for Culture, Media & Sport. Your data will only be reported in anonymous aggregated forms and will always be processed in accordance with the Data Protection

Act 2018 and therefore also in accordance with the General Data Protection Regulation (GDPR).

As part of the Creative Informatics programme we are monitoring the diversity of participants to ensure we are reaching and serving appropriate diverse audiences. As this information is sensitive and personal these questions are optional, but (where provided) this data does contribute to our ongoing review and improvement of our programme. We will always ensure that equality and diversity monitoring data is reported anonymously and with respect for your privacy. If you prefer not to provide this information, please indicate this in the 'Prefer not to answer' box for the appropriate question(s).

To withdraw your answers: email suzanne.black@ed.ac.uk

Consent:

By proceeding with the study, I agree to all of the following statements:

- I have read and understood the above information.
- I understand that my participation is voluntary, and I can withdraw at any time.
- I consent to my anonymised data being used in academic publications and
- presentations.
- I allow my data to be used in future ethically approved research.

1. I agree: ✱ *Required*

- ○ Yes
- ○ No

Note

This survey is designed to cover many aspects of Creative AI / machine learning, in Scotland and further afield and, as such, not all of the questions may be relevant to you.

Therefore, some of the questions are optional. Please answer the questions that best apply to you and skip those that are not relevant to your circumstances.

About you

In this section, we would like to find out a little about you.

2. What is your name?

3. What is the name of your company / business / organisation (if you have one)?

4. 4. Do you work in the creative industries? (for example, advertising, architecture, art, crafts, design, fashion, film, music, performing arts, publishing, R&D, software, toys and games, TV and radio, and video games). ✱ *Required*

- ☐ In a professional capacity
- ☐ In a voluntary capacity
- ☐ As a teacher / educator
- ☐ Does not apply
- ☐ Other

4.a. If you selected Other, please specify:

5. Where are you based? ✱ *Required*

- Scotland
- Rest of UK
- Rest of Europe
- Further afield

Experience with AI

For the purposes of this survey, we define ‘Creative AI’ broadly to include any use of machine learning (ML) systems at any stage in the creative process. These may include Generative Adversarial Networks (GANs), Large Language Models (LLMs), and speech, image, text and audio recognition and generation.

6. What is your relationship to Creative AI / Machine Learning? ✱ *Required*

- ☐ I do not pay any attention to it
- ☐ I follow news and updates about it
- ☐ I have played around with it
- ☐ I use it extensively in work or research
- ☐ I consider myself to be an expert in it
- ☐ Other

6.a. If you selected Other, please specify:

7. Do you have experience in using Creative AI / Machine Learning? ✱ *Required*

- ◯ Lots of experience
- ◯ Some experience
- ◯ A little experience
- ◯ No experience

Potential for use of AI

8. Do you plan to use Creative AI / Machine Learning in the future to help you in your work or artistic practice? If so, please tell us about your plans. (We will treat these as commercially confidential.) ✱ *Required*

9. What are the barriers preventing you from working with Creative AI / Machine Learning? ✱ *Required*

- ☐ Access to tools / software
- ☐ Concerns around privacy / ethics / legality
- ☐ Confidence
- ☐ Funding
- ☐ Interest
- ☐ Skills
- ☐ Time
- ☐ Training
- ☐ Other

9.a. If you selected Other, please specify:

10. Do you engage in creative practive where you do not use AI tools / Machine Learning? Please tell us about that. ✱ *Required*

Experience with AI, contd.

In this section, we would like to find out about your experience with Creative AI / Machine Learning.

11. I am confident in using Creative AI / Machine Learning: ✱ *Required*

- Strongly Agree
- Agree
- Neither Agree Nor Disagree
- Disagree
- Strongly Disagree

12. Please describe your Creative AI / Machine Learning work to date. ✱ *Required*

13. How did you get started using Creative AI / Machine Learning in your work?

14. Have you produced anything using Creative AI / Machine Learning. Please tell us about this work.

15. Please tell us about any plans you have for future Creative AI / Machine Learning projects. (We will treat these as commercially confidential.)

16. Are there any barriers preventing you from doing more with Creative AI / Machine Learning? ✱ *Required*

- ☐ Access to tools / software
- ☐ Concerns around privacy / ethics / legality
- ☐ Confidence
- ☐ Funding
- ☐ Interest
- ☐ Skills
- ☐ Time
- ☐ Training
- ☐ Does not apply
- ☐ Other

16.a. If you selected Other, please specify:

17. Do you have knowledge of / experience with / an interest in AI / Machine Learning for music / audio in Scotland? ✱ *Required*

- ○ Yes
- ○ No

Funding and Support

In this section, we would like to find out about sources of funding or support you have used or are aware of.

18. Below is a list of organisations that offer funding or support for Creative AI and Machine Learning. Please select the ones you are aware of. (For more information on these see Bayes AI Accelerator, Bridge AI, Creative Scotland, Innovate UK, Open Data Institute.) ✱ *Required*

- ☐ Bayes AI Accelerator
- ☐ Bridge AI
- ☐ Creative Scotland
- ☐ Innovate UK
- ☐ Open Data Institute
- ☐ None of these
- ☐ Other

18.a. If you selected Other, please specify:

19. Have you previously been awarded funding for innovation or R&D in Creative AI / Machine Learning from the following: ✱ *Required*

- ☐ Bayes AI Accelerator
- ☐ Bridge AI
- ☐ Innovate UK

- [ ] Creative Scotland
- [ ] Open Data Institute
- [ ] Does not apply
- [ ] Other

19.a. If you selected Other, please specify:

20. If you had access to funding, what Creative AI / Machine Learning projects would you like to do?

21. What minimum level of funding would enable you to undertake the work you would like to do?

22. Are there any barriers to getting the funding you need for Creative AI / Machine Learning projects? ✱ *Required*

- ☐ Application forms are too detailed
- ☐ Available funding opportunities are not suited to my needs
- ☐ Not aware of funding opportunities
- ☐ Too few funding opportunities
- ☐ Does not apply
- ☐ Other

22.a. If you selected Other, please specify:

Training and other resources

In this section, we would like to find out about sources of training or other resources you may have used.

23. Have you used any of these tools / software / processes in creative work? ✱
Required

- ☐ AIVA
- ☐ Amadeus Code
- ☐ Bard (Google)
- ☐ Character.AI
- ☐ ChatGPT (OpenAI)
- ☐ Claude
- ☐ Craiyon
- ☐ DALL-E (OpenAI)
- ☐ Ecrett
- ☐ Generative adversarial networks (e.g. CycleGAN)
- ☐ GPT-4 (OpenAI)
- ☐ Infinite Album
- ☐ Lalal.ai
- ☐ Magenta Studio
- ☐ Midjourney
- ☐ Murf
- ☐ MuseNet (OpenAI)
- ☐ Orb Produce Suite
- ☐ Podcastle
- ☐ Soundful
- ☐ Soundraw
- ☐ Stable Diffusion
- ☐ None

☐ Other

23.a. If you selected Other, please specify:

23.b. If you have used any, please tell us in what ways.

24. Have you undertaken any training in AI / Machine Learning? ✱ *Required*

○ Yes

○ No

24.a. Please tell us about this training (when the training took place, where from, etc.)

25. Are there any barriers preventing you from accessing training about Creative AI /

Machine Learning? ✱ *Required*

- ☐ Can’t find the training I want
- ☐ Can’t find training at an appropriate skill level
- ☐ Don’t know where to find information on training
- ☐ Don’t know what training I should do
- ☐ Feel overwhelmed by all the different options for training
- ☐ Lack of funding to pay for training
- ☐ Not enough time to do training
- ☐ Training I want requires existing knowledge (e.g. coding, art skills)
- ☐ Other

25.a. If you selected Other, please specify:

26. Are there any Creative AI / Machine Learning tools / software / processes that you would like to know more about? Please tell us which ones.

27. Are there any other resources you require to undertake Creative AI / Machine Learning projects (for example, collaborators, skills, tools, computing technology)?

28. Where do you go to find out about Creative AI / Machine Learning tools and resources? ✱ *Required*

- [ ] Colleagues
- [ ] Creative Organisations (like Creative Edinburgh)
- [ ] Educational organisations (like universities)
- [ ] Following individuals (like industry or creative practitioners) on social media
- [ ] Friends
- [ ] Meet-up groups or other peer communities
- [ ] News media
- [ ] Online courses (like MOOCs, Skillshare, etc.)
- [ ] Professional bodies (like the Scottish Music Industry Association) or Membership bodies (like Applied Arts Scotland)
- [ ] Self-led internet resources (like GitHub, Stack Overflow, toolkits etc.)
- [ ] Social media
- [ ] Technology groups (like the Scottish AI Alliance)
- [ ] None
- [ ] Other

28.a. If you selected Other, please specify:

29. Is there inspiring work using Creative AI / Machine Learning, or support for it, that you know of outside of Scotland?

Thoughts around AI

In this section we would like to find out about your thoughts on Creative AI / Machine Learning.

30. Please tell us about why you are interested (or not!) in Creative AI / Machine Learning in your creative work / practices. ✱ *Required*

31. What do you think are the potential benefits of Creative AI / Machine Learning for creative work / practices? ✱ *Required*

32. What concerns do you have about Creative AI / Machine Learning? (Perhaps relating to ethics, Intellectual Property rights, Equality, Diversity & Inclusion, how well AI is understood?) ✱ *Required*

32.a. What support and advice do you think is needed to address these concerns?

32.b. Is this support and advice already available, as far as you know?

33. How do you check that Creative AI / Machine Learning tools are consistent with your values?

Thoughts around AI, contd.

In this section, we would like to find out about specifically about creative AI / Machine Learning in Scotland.

Please skip these questions if they are not relevant to you.

34. Are you aware of any communities or initiatives making good use of AI / Machine Learning in Scotland. What are they?

35. Have you seen any projects working in AI / Machine Learning in Scotland that you would recommend we look at?

36. Is there anything about the Scottish context which changes how we should be thinking about AI / Machine Learning in this region?

37. How hard do you think it is to be able to innovate in Creative AI / Machine Learning in Scotland?

38. From your experience, what type of interventions should we make to encourage creative use of AI / Machine Learning in Scotland? (For example, funding, training, access to opportunities.)

39. Is there anything specific to AI / Machine Learning in music / audio in Scotland that you think is important for us to know? Please tell us.

Final thoughts

40. Is there anything else you could tell us about how best to support your ambitions in Creative AI / Machine Learning in general, or Creative AI / Machine Learning and music / audio?

About You

As part of the Creative Informatics programme we are monitoring diversity to ensure we are reaching and serving appropriate diverse audiences.

We will always ensure that equality and diversity monitoring data is reported anonymously and with respect for your privacy.

If you prefer not to provide this information, please indicate this in the 'Prefer not to answer' box for the appropriate question(s).

41. Please indicate your age range:

- 18 - 24
- 25 - 35
- 35 - 44
- 45 - 54
- 55 - 64
- 65+
- Prefer not to answer

42. Nationality/Ethnicity:

- White
- Mixed/Multiple Ethnic Group
- Asian or Asian British
- Black/African/Caribbean/Black British
- Other Ethnic Groups
- Prefer not to answer

43. I identify my Gender as:

____

44. Sexual Orientation:

- ○ Heterosexual
- ○ Bisexual
- ○ Gay
- ○ Lesbian
- ○ Other
- ○ Prefer not to answer

45. Please indicate if you identify as having a disability:

- ○ Yes
- ○ No
- ○ Prefer not to answer

Mailing List and Prize Draw

46. What is your email address? ✱ *Required*

46.a. Do you grant permission to Creative Informatics to use this email address to contact you in relation to this survey and this project? ✱ *Required*

- Yes
- No

46.b. Would you like to be added to the Creative Informatics mailing list, in order to hear about any follow up activities, including Creative AI opportunities?

- Yes
- No

46.c. Do you grant permission for this email to be used to enter you into a prize draw for one of five £50 Love2Shop gift cards as a thank you for taking part in this survey? ✱ *Required*

- Yes
- No

Final page

Thank you for completing this research survey for the Creative AI Demonstrator Project. Your answers will help us to make recommendations about the landscape of Creative AI and Machine Learning in the UK and the resources needed to grow this field.

If you would like to discuss any further aspects of this survey, please contact the collecting researcher, Dr Suzanne Black, on suzanne.black@ed.ac.uk.

We will share the survey responses at future project events, and hope to see you there!
